# Cetuximab associated scrotal ulcers in the setting of metastatic colorectal cancer

**DOI:** 10.1016/j.jdcr.2024.08.001

**Published:** 2024-08-30

**Authors:** Neha Arora, Sanober Amin

**Affiliations:** a Texas A&M University School of Medicine, Dallas, Texas; b Dermatology Solutions, Grapevine, Texas

**Keywords:** cetuximab therapy, drug eruption, genital ulcers

## Introduction

Cetuximab is a monoclonal antibody inhibitor to the epidermal growth factor receptor (EGFR).^1^ Its mechanism of action is through blockage of the EGFR signaling pathway, which plays a crucial role in cell differentiation, proliferation, and angiogenesis.^1^ Cetuximab is Food and Drug Administration approved for use in certain types of colorectal cancer as well as for head and neck squamous cell carcinomas.^2^ Off-label uses for this drug include EGFR-expressing non-small cell lung cancer and unresectable squamous cell skin cancer.^2^ Adverse effects from cetuximab therapy include headache, diarrhea, infection, and various cutaneous manifestations such as acneiform eruptions, paronychial inflammation, cellulitis, and hypertrichosis.^3^ Here we present a case of cetuximab-associated scrotal ulcers in a 43-year-old man with metastatic colorectal cancer.

## Case

**Fig 1:**
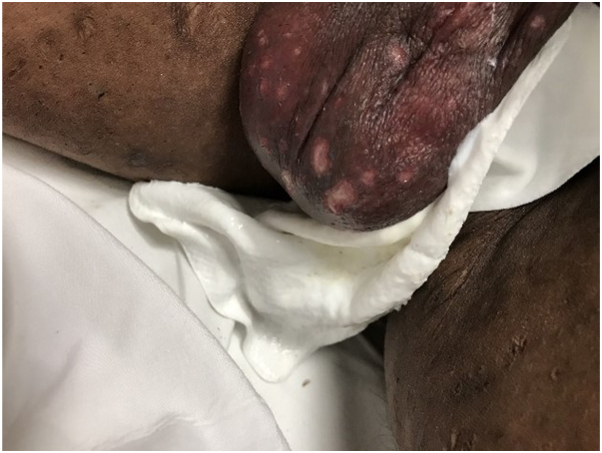
Ulcers on scrotal skin.

**Fig 2:**
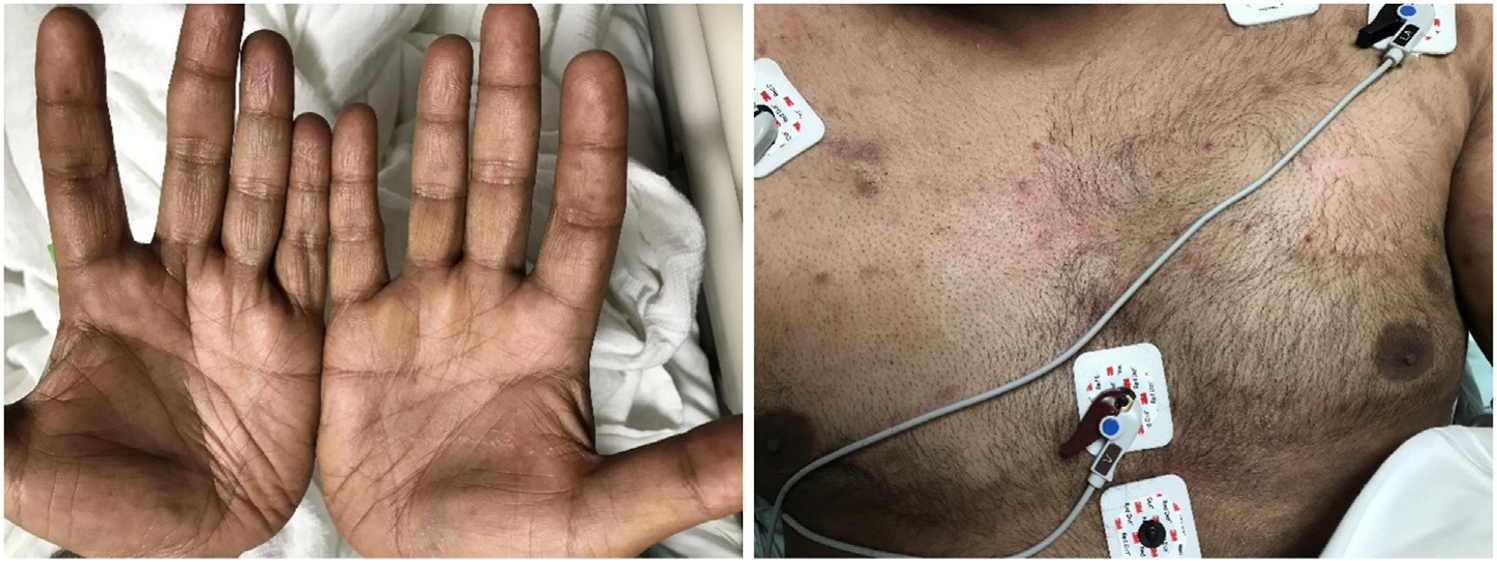
Hyperpigmented macules seen on palms and anterior chest.

A 43-year-old male with colorectal cancer undergoing cetuximab therapy presented with new-onset scrotal ulcers (Fig 1) and hyperpigmented macules on the palms, soles, and anterior chest wall (Fig 2). The patient was started on cetuximab for cancer treatment 6 weeks prior. Ulcers were present exclusively on the scrotum (no mucosal involvement) and were extremely painful. Hyperpigmented macules arose at the same time as the ulcers. A shave biopsy of the scrotal ulcers was performed to establish histopathological diagnosis, revealing interface dermatitis with eosinophils. Immunostaining for herpes simplex virus 1 and 2, varicella zoster virus, Treponema pallidum, and fungi was negative. Given the presence of eosinophils on pathology, the diagnosis of drug eruption was favored. Cetuximab was subsequently discontinued, leading to resolution of both scrotal ulcers and hyperpigmented macules.

## Discussion

We present the case of a 43-year-old man who developed scrotal ulcers and hyperpigmented macules on the palms and anterior chest after initiating cetuximab therapy for metastatic colorectal cancer. Both ulcers and macules resolved after cessation of cetuximab.

The management of dermatologic adverse effects caused by molecularly targeted cancer agents is reported to cost an estimated $1920 per patient.^4^ These cutaneous manifestations can impact quality of life, drug adherence, and psychological well-being. The most common dermatologic finding associated with cetuximab is a papulopustular acneiform exanthem seen in 80% to 86% of patients.^5^ Other reported cutaneous findings include xerosis, eczema, hyperpigmentation, pruritis, and hair changes (which can present as both hypertrichosis and alopecia).^3^^,^^5^

While palmoplantar eruptions and hyperpigmentation have been observed, genital skin lesions or scrotal ulcer drug eruptions are very rarely reported with cetuximab use.^6^ It remains unclear whether this reaction is directly caused by EGFR inhibition or if it is idiosyncratic.^7^ In patients presenting with sole genital involvement, these ulcers may raise suspicion for an infectious etiology—potentially delaying diagnosis and appropriate treatment. Given appropriate historical and clinical indicators, patients on cetuximab who develop genital ulcers may need evaluation for drug eruption.

## Conflicts of interest

None disclosed.
